# Text-based similarity searching for hit- and lead-candidate identification

**DOI:** 10.1186/1758-2946-4-S1-O12

**Published:** 2012-05-01

**Authors:** Volker Hähnke

**Affiliations:** 1 National Center for Biotechnology Information, National Library of Medicine, National Institutes of Health, Department of Health and Human Services, 8600 Rockville Pike, Bethesda, MD 20894, USA

## 

The Pharmacophore Alignment Search Tool (PhAST) is a string-based approach to virtual screening. Molecules are represented by linear sequences which describe their respective pattern of interaction possibilities. The problem of molecule linearization is tackled by applying Minimum Volume Embedding in combination with a Diffusion Kernel to the molecular graph [1,2]. Linear representations are compared using global pairwise sequence alignment [3]. PhAST exhibited enrichment capabilities comparable or superior to most common virtual screening approaches. Compound rankings were proven to be dissimilar to those of other virtual screening techniques. It was shown that emphasis on key interactions through the application of position specific weights in the alignment process significantly increases enrichment.

Significance of chemical similarity was determined in form of p-values of global alignment scores, calculated in an approach that was adapted from its original application to local sequence alignments of protein sequences utilizing Marcov chain Monte Carlo simulation [4]. Bonferroni correction was used to correct p-values with respect to the size of the screening library [5].

PhAST was employed in two prospective applications: A screening for non-nucleoside analogue inhibitors of bacterial thymidine kinase yielded a hit with a distinct structural framework but only weak activity. Screenings for drugs that are not members of the NSAID (non-steroidal anti-inflammatory drug) class as modulators of gamma secretase resulted in a potent modulator with clear structural distinction from the reference compound.
